# Engineering long shelf life multi-layer biologically active surfaces on microfluidic devices for point of care applications

**DOI:** 10.1038/srep21163

**Published:** 2016-02-17

**Authors:** Waseem Asghar, Mehmet Yuksekkaya, Hadi Shafiee, Michael Zhang, Mehmet O. Ozen, Fatih Inci, Mustafa Kocakulak, Utkan Demirci

**Affiliations:** 1 Bio-Acoustic-MEMS in Medicine (BAMM) Laboratory, Department of Radiology, Canary Center at Stanford for Cancer Early Detection, Stanford University School of Medicine, Electrical Engineering Department (by courtesy), Stanford University, Palo Alto, CA 94304; 2 Department of Electrical & Computer Engineering and Computer Science, Florida Atlantic University, Boca Raton FL, 33431; 3 Bio-Acoustic-MEMS in Medicine (BAMM) Laboratory, Division for Biomedical Engineering, Renal Division, Department of Medicine, Brigham and Women’s Hospital, Harvard Medical School, Cambridge, MA, 02139, USA; 4 Başkent University, Faculty of Engineering, Biomedical Engineering Department, Ankara, Turkey

## Abstract

Although materials and engineered surfaces are broadly utilized in creating assays and devices with wide applications in diagnostics, preservation of these immuno-functionalized surfaces on microfluidic devices remains a significant challenge to create reliable repeatable assays that would facilitate patient care in resource-constrained settings at the point-of-care (POC), where reliable electricity and refrigeration are lacking. To address this challenge, we present an innovative approach to stabilize surfaces on-chip with multiple layers of immunochemistry. The functionality of microfluidic devices using the presented method is evaluated at room temperature for up to 6-month shelf life. We integrated the preserved microfluidic devices with a lensless complementary metal oxide semiconductor (CMOS) imaging platform to count CD4^+^ T cells from a drop of unprocessed whole blood targeting applications at the POC such as HIV management and monitoring. The developed immunochemistry stabilization method can potentially be applied broadly to other diagnostic immuno-assays such as viral load measurements, chemotherapy monitoring, and biomarker detection for cancer patients at the POC.

Although methods to biopreserve proteins and antibodies using polyols such as sugar solutions, sorbitol, glycerol, and various polymers have been well-established^1^,^2^,^3^,^4^,^5^,^6^, keeping surfaces of microfluidic devices engineered with multi-layer surface chemistry functional remains a challenge^7^,^8^. Multi-layer surface chemistry has been shown to lead to better antibody orientation and higher capture efficiency^9^. In multi-layer surface preservation, antibody conjugation along with surface functional groups needs to be stabilized. Stabilization of immuno-functionalized microfluidic devices will address challenges associated with delivering healthcare in resource-constrained settings lacking reliable electricity and refrigeration^10^,^11^,^12^,^13^,^14^,^15^, and having limited access to state-of-the-art medical equipment. Although microfluidic-based devices have enabled point-of-care (POC) testing in disease diagnostics including CD4 T cell counts and viral load measurements^14^,^16^,^17^,^18^,^19^,^20^,^21^, these devices need to be stored at low temperatures (4–8 ^o^C) to prevent denaturation of capture antibodies and loss of function^22^,^23^. Transportation of biomaterials in cold chain is expensive, thus significantly increase the assay cost. In addition to the issues related to cost cold storage may not even be available in resource limited settings. To address these challenges, there is a need to preserve multi-layer immuno-functionalized microfluidic devices in refrigeration-free settings.

Although various methods has been available to preserve antibodies in dry or solution form such as freezing, drying, lyophilization, and dilution^24^,^25^, stabilization of functionalized microfluidic devices is recently being investigated^26^,^27^,^28^. Unlike, lateral flow and dipstick assays, where single antibodies are stabilized, microfluidic devices can have multiple antibodies/proteins conjugated together as multi-layers and immobilized inside microfluidic channels. Multi-layer surface chemistry achieves better orientation of antibodies for higher and repeatable capture efficiencies^9^. Herein, we investigated the utilization of trehalose for preserving complex multi-layer surface chemistry in microfluidic channels at refrigeration-free conditions. In nature, trehalose is present in unicellular organisms as a stress-responsive factor^29^,^30^,^31^. These organisms synthesize trehalose that helps to retain their cellular integrity under stress conditions such as heat, desiccation, and cold^29^,^30^,^31^. Although trehalose has been extensively explored in preservation and stabilization of biological molecules and cells^32^,^33^,^34^,^35^,^36^, it has not been investigated to preserve multi-layered immune-functionalized microfluidic surfaces to potentially store these surfaces at room temperature. Using trehalose as a naturally occurring stabilization agent, we present a method to preserve multi-layered surfaces of immuno-functionalized microfluidic devices with immobilized antibodies for broad applications in POC diagnostics, achieving long term storage up to 6 months at room temperature. Initially, we developed a stabilized microfluidic device for CD4 T cell counting using unprocessed whole blood samples. The stabilized and dried microfluidic devices are stored at room temperature and can be used for CD4 T cell counting at the time of need by simply re-activating the surfaces using a PBS wash. We have also integrated these stabilized microfluidic devices post-reactivation with a lensless imaging technology^12^,^37^, where captured CD4 T cells are counted rapidly and automatically from unprocessed whole blood creating a portable, battery-operated, inexpensive, and microscope-free CD4 T cell counting platform with a long shelf-life enabling applications at POC for resource-limited, primary care, or bedside settings^12^,^37^.

## Results

### Microfluidic device assembly and surface functionalization

**Figure 1:**
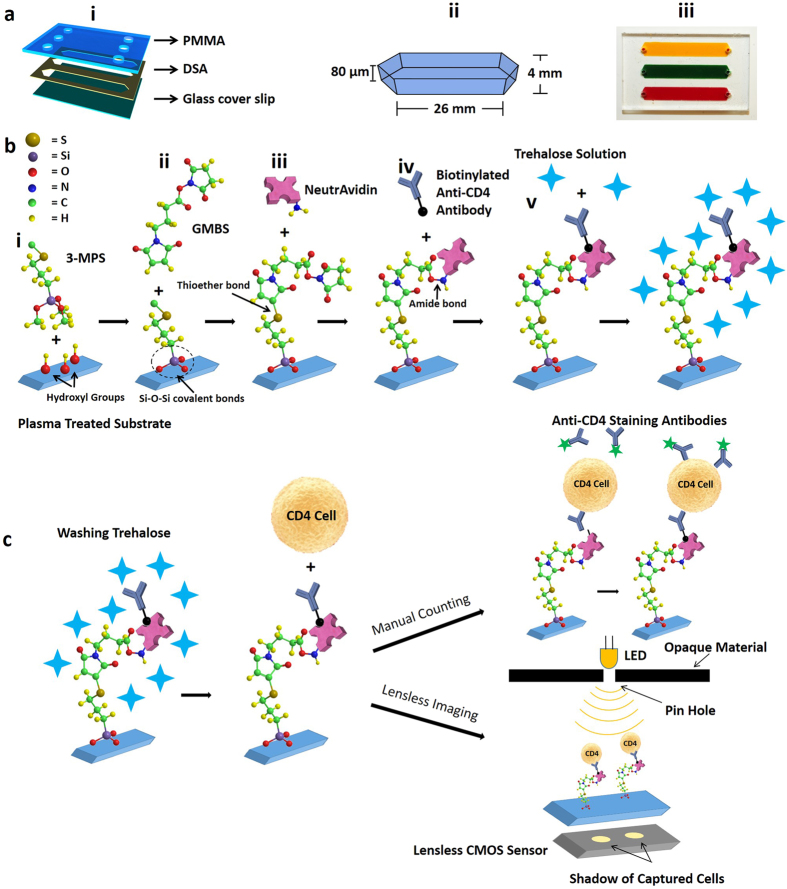
Microfluidic device assembly, surface functionalization, and CD4 T cells counting. (**a**) (i) 3D schematic of a three channel microfluidic device fabricated by poly methyl methacrylate (PMMA), double sided adhesive (DSA) and glass cover slip. (ii) Physical dimensions of a channel. (iii) Actual image of a microfluidic device where channels are filled with food dyes and blood. (**b**) NeutrAvidin-Biotin based antibody immobilization and trehalose preservation. Oxygen plasma treated cleaned glass covers slips were silanized with 3-MPS followed by GMBS treatment. (i) During the silanization step, three methoxy (CH3O-) groups of 3-MPS are replaced with 3 hydroxyl groups (OH-) on the surface, thus forming strong covalent -Si-O-Si- bonds. (ii) GMBS is an amine-to-sulfhydryl crosslinker that contains NHS-ester and maleimide reactive groups at opposite ends of a short spacer arm. Maleimide group react with -SH groups at 3-MPS, forming stable thioether linkages. (iii) NHS ester end of GMBS couples with amines (located at Neutravidin) to form stable amide bonds. (iv) Surface was further functionalized biotinylated anti-CD4 antibody. (v) Finally, trehalose solution was used to protect the antibodies from degradation. (**c**) After washing trehalose, whole blood was applied to the channels. Devices were washed, and cells were stained with DAPI for all WBCs, and Alexa Flour 488 conjugated anti-CD4 antibody for CD4 T cells. The unstained captured CD4 T cells can automatically be counted using lensless complementary metal-oxide semiconductor (CMOS) sensor.

To present a trehalose-based approach for the preservation of multi-layer surface chemistry on channel surfaces, we fabricated a microfluidic device and constructed multi-layer surface chemistry (Fig. 1). Microfluidic device was designed and assembled by assembling a layer of double-sided adhesive (DSA) with poly methyl methacrylate (PMMA) and glass cover slip (Fig. 1a). The devices were functionalized with anti-CD4 antibody as explained in Methods section (Fig. 1b). Functionalized devices were stabilized using a trehalose solution followed by a drying process to preserve them at room temperature in a vacuum-sealed plastic bag (Methods section). These devices were used for capturing CD4 T cells from whole blood. Captured CD4 T cells were counted using a fluorescence microscope and lensless complementary metal-oxide semiconductor (CMOS)^38^ sensor (Fig. 1c).

### Device drying, stabilization, storage and reactivation

**Figure 2:**
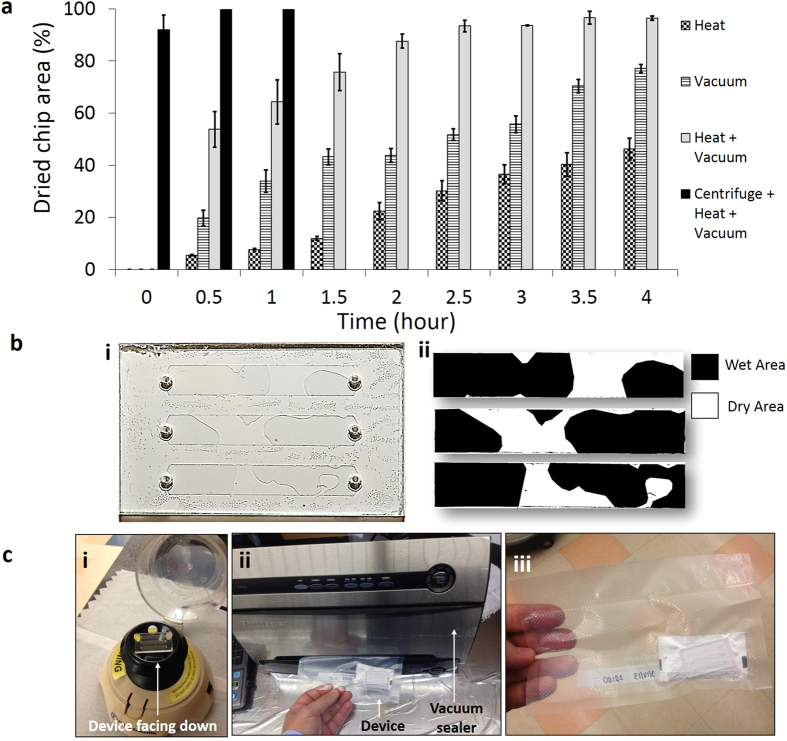
Microfluidic device drying, packing and storing methods. (**a**) Devices were dried by four different methods, (i) heating (37 ^o^C), (ii) vacuum (−761 mmHg), (iii) heating and vacuum (37 ^o^C + −761 mmHg), and (iv) device centrifugation followed by heating and vacuum (37 ^o^C + −761 mmHg), and dried device surface area was measured to quantify the drying efficiency. By using only heating or vacuum, devices were not dried fully even after 4 hours. Using heating plus vacuum we were able to dry 96% of the device surface area in 4 hours. The most time efficient method was device centrifugation followed by heating and vacuum. Using this method, devices were dried fully in an hour, n (no. of channels) =3. Error bars represent standard deviation. (**b**) (i) Whole device images were taken to quantify the drying area. Channel areas were cropped from image and drying area was calculated using *ImageJ* software. (ii) White and black areas represent the dried and wet regions of the device surface. (**c**) (i) Microfluidic devices were centrifuged to remove fluid from channels for fast drying. The device was placed on the centrifuge upside down (channel inlets facing downwards). To remove the remaining fluid, vacuum heating was applied. (**ii**) Devices were inserted into plastic bags with drying agent. Air inside the bag was pumped out using vacuum sealer and bag was sealed. (iii) Devices were ready for storing at room temperature.

Functionalized microfluidic devices stabilized using trehalose cannot be efficiently dried at room temperature as channel inlet and outlet are small (0.6 mm diameter), which does not allow trehalose solution to evaporate quickly. Four methods were employed to dry the trehalose-treated functionalized microfluidic devices, (i) heating (37 ^o^C), (ii) vacuum (−761 mmHg), (iii) heating and vacuum (37 ^o^C + −761 mmHg), and (iv) device centrifugation followed by heating and vacuum (37 ^o^C + −761 mmHg) (Fig. 2a, n (number of channels tested) =3 for each method). To quantify the dried device surface area, we used *ImageJ* software (National Institute of Health, http://rsbweb.nih.gov/ij/) as shown in Fig. 2b, where white and black areas represent the dried and wet regions of the chip surface. Heating or vacuum alone was not able to dry devices faster, only 46.2 ± 4.1% (heating) and 77.1 ± 1.63% (vacuum) of device areas were dried in 4 hours (Fig. 2a). Vacuum plus heating provided better results than that of vacuum or heating alone as 96.4 ± 0.87% of device area was dried in 4 hours. To decrease the device drying time, we developed a method to first remove the excessive fluid inside microfluidic devices by centrifugation, and then vacuum plus heating was applied to dry the surface (Fig. 2c). Device centrifugation at 6600 rpm for 5 seconds removed 92 ± 5.5% of the fluid (Methods section). Remaining fluid was dried using vacuum plus drying. It took 30 minutes to fully dry the trehalose-treated functionalized microfluidic devices by a centrifugation-based method (Fig. 2a). The trehalose-treated dried devices were sealed in a plastic vacuum bags along with Silica gel Rubin drying bags (Fig. 2c). The functionality of trehalose-treated dried devices was then reactivated by PBS wash to remove trehalose from microfluidic channels.

### Testing of dried microfluidic devices and lensless imaging

**Figure 3:**
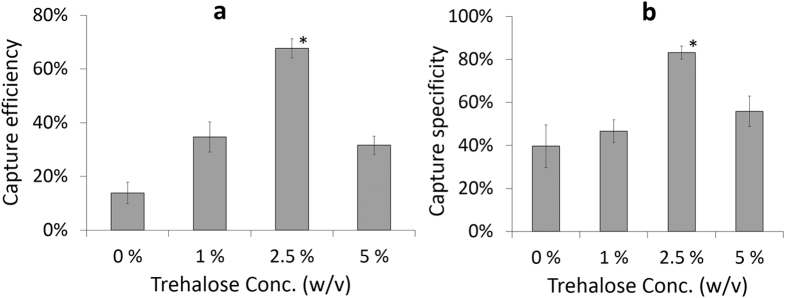
Optimization of trehalose concentration for stabilization of surface chemistry inside microfluidic channels. Comparison of CD4 T cell (**a**) capture efficiency and (**b**) capture specificity at different trehalose concentrations (w/v); 0%, 1%, 2.5%, and 5%. The devices were preserved for 2 weeks at room temperature before testing (*p < 0.05 between devices preserved at 2.5% trehalose (w/v) and other concentrations, n = 3).

**Figure 4:**
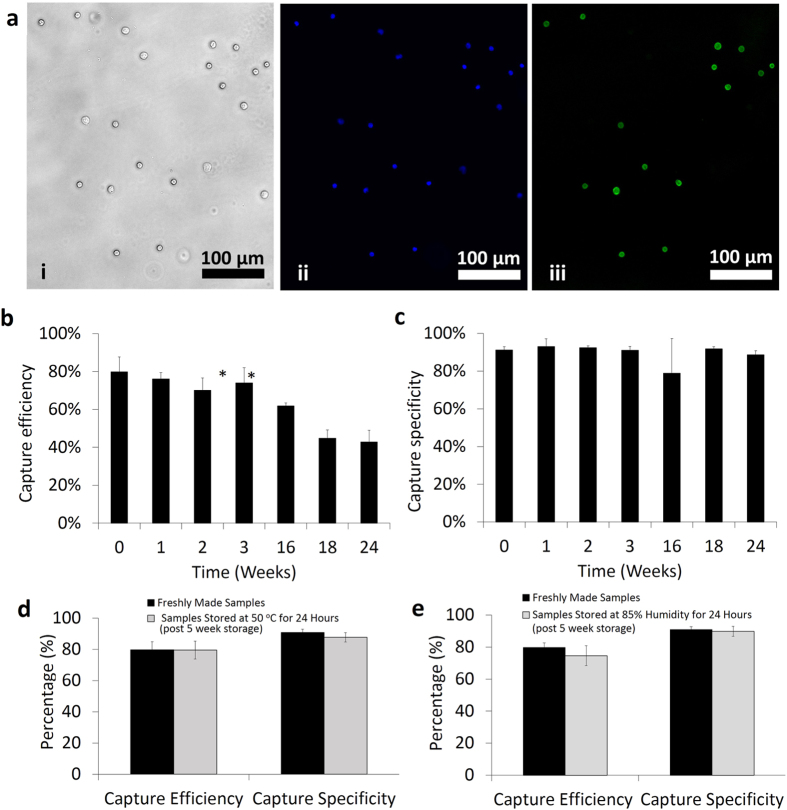
Testing of stabilized microfluidic devices. (**a**) Microscope images of captured cells inside a channel, (i) bright field image, (ii) DAPI stained cells (nucleated cells), and (iii) CD4 T cells. CD4 T cell capture efficiency (**b**) and capture specificity (**c**) were calculated over a period of 6 months (*p < 0.05 between freshly prepared devices and dried devices, n = 3–6). Error bars represents standard deviation. (**d**) Comparison of CD4 T cell capture efficiency and specificity between freshly functionalized devices and devices preserved for 5 weeks at room temperature followed by storage at 50 ^o^C for 24 hours. (**e**) Comparison of CD4 T cell capture efficiency and specificity between freshly functionalized devices and devices preserved for 5 weeks at room temperature followed by storage at 85% humidity for 24 hours.

Before running the cell capture experiments, a microfluidic device washing protocol was optimized. Experimental findings suggested that microfluidic devices should be washed three times with PBS to remove unbound blood cells from the channels (Supplementary Figure 1). To optimize the trehalose concentration for surface chemistry stabilization, trehalose solutions at different concentrations (1–5% (w/v)) were used. Negative control sample was determined as surfaces without trehalose treatment. Stabilized and dried devices were kept at room temperature for 2 weeks before testing for CD4 T cell capture efficiency and specificity from whole blood. Without trehalose treatment (negative control sample: 0% v/w), CD4 T cell capture efficiency and specificity were observed lower than the other trehalose concentrations (Capture efficiency at negative controls = 13.9% ± 4.0%, specificity at negative controls = 39.7% ± 9.9%). We observed a significant increase in CD4 T cell capture efficiency and specificity when 2.5% (w/v) trehalose was employed (Fig. 3a,b), compared to other concentrations; 0% (negative control), 1%, and 5%. At a higher trehalose concentration (5%), CD4 T cell capture efficiency and specificity started decreasing compared to 2.5%. As a result, higher trehalose concentration may interfere with the recognition sites of antibody to the target molecule/epitope as previously reported^26^. Based on these observations, we continued experiments with 2.5% trehalose. Trehalose-treated dried microfluidic devices were also tested for capturing CD4 T cells from whole blood after 1, 2, 3, 16, 18, and 24 weeks. Captured cells were stained with DAPI and anti-CD4-Ab-conjugated-AlexaFlour-488 dyes for counting nucleated (WBCs) and CD4 T cells respectively (Fig. 4a). CD4 T cell capture efficiency was calculated by captured CD4 T cells divided by total CD4 T cells injected into the microchannels, whereas CD4 T cell capture specificity was calculated by dividing AlexaFlour-488 stained cells by DAPI stained cells (Methods section). CD4 T cell capture efficiency changed from 79.9 ± 7.8% (freshly prepared devices) to 76.1 ± 3.4%, 70.1 ± 6.4%, 74.0 ± 7.9%, 61.9 ± 1.5%, 44.8 ± 4.5%, and 42.8 ± 6.3% for trehalose-treated devices tested after weeks 1, 2, 3, 16, 18, and 24, respectively (Fig. 4b, n = 3–6 for each time point). CD4 T cell capture efficiency remained above 60% after 4 months while it decreased significantly to 42.8% at the end of 6^th^ month. The possible reason for this reduction in capture efficiency after 4 months was due to the fact that the sealed plastic bag started losing vacuum. CD4 T cell capture specificity remained almost stable over a period of 6 months with specificity values of 91.2 ± 1.8% (freshly prepared devices), and 88.6 ± 2.2% for trehalose-treated dried functionalized devices tested after 6 months (Fig. 4c). We have also tested the preserved devices at higher temperature (50 °C) and higher humidity (85%) to mimic environmental conditions in resource-constrained settings. Thus, we have stored the devices at room temperature for 5 weeks and then heated them at 50 ^o^C for 24 hours using hotplate. The devices remained sealed in bags during the heating. Devices were cooled down back to room temperature before evaluation. These preserved devices resulted in a CD4 T cell capture efficiency and specificity of 79.5 ± 5.8% and 87.7 ± 3.0% respectively from whole blood (Fig. 4d). To evaluate humidity effect on device performance, devices that have been preserved for 5 weeks were stored inside an oven at 85% humidity and 30 °C for 24 hours. These preserved devices at higher humidity provided a CD4 T cell capture efficiency and specificity of 74.6 ± 6.2% and 89.8 ± 3.1% respectively, from whole blood (Fig. 4e). As a result, these findings were comparable to those of freshly functionalized devices (capture efficiency = 79.7 ± 5.2%, capture specificity = 90.9 ± 2.1%).

**Figure 5:**
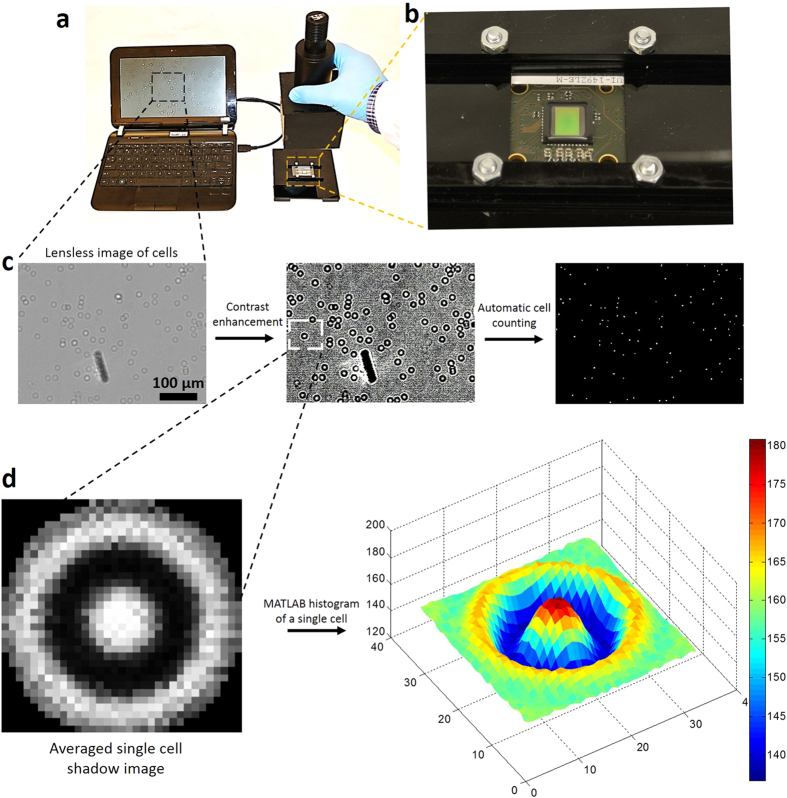
Lensless CMOS imaging platform for automatic counting of captured CD4 T cells from whole blood. (**a**) Setup for lensless imaging using CMOS sensor. Shadows of captured CD4 T cells were detected on CMOS sensor surface. CMOS sensor was connected to laptop for image acquisition. (**b**) Larger view of CMOS sensor and home-built plastic platform to hold sensor device fixed. (**c**) Shadows of captured cells were processed using developed MATLAB program to enhance contrast for better cell counting. (**d**) Averaged cell shadow image is generated by averaging the intensity histograms of 30 randomly selected images. The color bar shows the intensity values (arbitrary units), where red color shows high light intensity areas. Shadows of capture cells are compared with sample cell shadow for correlation and cells are counted automatically.

To develop an automatic CD4 T cell counting platform, we have integrated the CMOS lensless technology with microfluidic devices. Shadows of all captured cells were acquired on a CMOS sensor, due to its wide field of view within few seconds (Fig. 5). We have developed a MATLAB algorithm to automatically count the captured cell shadows (Supplementary Figure 2, Methods section). Averaged cell shadow image is generated by averaging the intensity histograms of 30 randomly selected cell shadows (Supplementary Figure 3, Fig. 5d). The algorithm uses an averaged cell shadow image and compares its intensity histogram with the image obtained from microfluidic channel. The areas showing correlation higher than the threshold value are counted as cells. In correlation method, only those shadows are identified as cells that are similar to the averaged cell shadow, while the shadows of debris in the microfluidic channel are neglected (Supplementary Figure 4, Fig. 5c,d). The algorithm uses the threshold value input by the user. Optimization of the threshold value is needed as lower threshold values would result in identifying non-cell shadows as cells (Supplementary Figure 5). This optimization of threshold value can be done easily by comparing identified cells (shown by “red” dots in Supplementary Figure 5) and cell shadows simultaneously. If debris is identified as a cell (“red” dot), there is a need to increase the threshold value as program is misidentifying noise as a cell at a specific threshold value. We have compared the counting efficiency of developed algorithm (for cell shadows) with microscope based manual cell counting and observed 97.4 ± 0.5% counting efficiency, when correct threshold values were used (Supplementary Table 2).

## Discussion

Although the microfluidic devices stabilized using the presented method show high capture efficiency and specificity, some of the devices lost vacuum over time. We have preserved 30 devices in total using this method and observed 6 devices lost their vacuum after a month, giving an overall process yield of 80%. We did not use these devices in our experiments due to potential loss of function due to the humidity in air that penetrates the channels over time under non-vacuum conditions. Drying process yield can be increased by using a vacuum sealer with temperature and vacuum controls, and using mylar metalized laminate bags that does not allow vapor and humidity to pass through^12^.

There are various mechanisms put forward to explain the mechanism by which trehalose stabilizes proteins such as vitrification, preferential exclusion and water replacement mechanisms^39^,^40^. According to vitrification theory, trehalose forms a glassy cocoon to physically protect protein from abiotic stresses^41^. For such a mechanism, the glass transition temperature (Tg) of a stabilizing agent should be higher than the storage temperature. The Tg of trehalose is 117 ^o^C, much higher than room temperature^41^. The better protein stabilizing ability of trehalose is due to its higher Tg compared to other sugar solutions such as sucrose or maltose^41^. Preferential exclusion mechanism proposes that trehalose isolates water molecules away from protein, which results reduction in protein radius, and hence, increases its compactness and stability^39^,^40^. According to exclusion theory, there is no direct interaction between protein and trehalose molecules^39^,^40^. Conversely, water replacement theory proposes that trehalose replaces water molecules by making hydrogen bonds with protein while drying^42^,^43^,^44^,^45^. The substitution of trehalose in place of water molecules maintains the three-dimensional structure of protein and stabilizes it in a dry form^42^,^43^,^44^,^45^. These mechanisms explain the stabilizing phenomena of trehalose, as it has been successfully used to preserve biomolecules, proteins, and cells^45^. Although trehalose was used by others for preserving mammalian cells (as a cryoprotective agent) and protein/antibody suspensions^32^,^33^,^34^,^35^,^36^, trehalose has not been investigated in preserving functionalized microfluidic devices with immobilized proteins and antibodies for refrigeration-free storage. Challenges associated with preserving a protein or cells in a suspension is completely different than preserving the biological surface with multiple layers of surface chemistry, which also has to meet diagnostic performance parameters of a medical device when brought into contact with another biological complex fluids such as whole blood. Here, we have used trehalose to stabilize the multi-layer surface chemistry composed of a complex structure of proteins and antibodies along with other functional materials such as 3MPS and GMBS in a microfluidic device.

CD4 T cell count has broad applications in chemotherapy monitoring, transplant patient monitoring and particularly in monitoring the efficacy of antiretroviral therapy (ART)^19^,^46^,^47^,^48^,^49^. For instance, according to the HIV treatment guidelines by World Health Organization (WHO), it is recommended to start the ART therapy when the individual’s CD4 cell count falls below 500 cells/μl^10^,^14^,^50^. Currently, due to lack of access to HIV testing, one in four people who started getting ART therapy had initial CD4 cell counts under 100 cells/μl which reflects late diagnosis, and, hence, higher risk of illness and death^10^. These global health challenges can be solved by developing diagnostic platforms, where testing can be done at the point of need.

In summary, microfluidic device drying and antibody stabilization method presented herein preserved the immuno-functionalized devices for 6 months in refrigeration-free settings. These preserved devices can be used for CD4 T cell counting at the point of need from a drop of unprocessed blood. The presented microfluidic device stabilization method is potentially widely applicable to other global health applications at resource-limited settings such as viral load, sepsis, tuberculosis, malaria, and cancer detection^9^,^16^,^51^,^52^,^53^,^54^,^55^,^56^,^57^,^58^,^59^,^60^,^61^,^62^,^63^,^64^,^65^. It would also have potential applications at the developed world settings such as at a primary care physician’s office or at home settings at the point of living. The integration of lensless CMOS imaging technology with microfluidic devices offers opportunities to count selectively captured cells from whole blood without any fluorescent labels. Lensless imaging sensor provides an ultra wide field-of-view to analyze the whole microfluidic device in few seconds. CMOS camera of a commercialized cell phone can be utilized to count cells automatically, and it can substitute the CMOS sensor presented here. Future work will benefit from testing of the preserved microfluidic devices at varying temperature ranges and humidity levels as well as in a resource-constrained settings in the developing world.

## Methods

### Materials and Reagents

For microfluidic device fabrication, a 3.175 mm thick clear cast acrylic sheet (PMMA, Polymethymetacrylate, 8560K239) and a 80 μm thick optically clear double sided adhesive (DSA, CN: 8113) were purchased from McMaster Carr Supply Co. Inc (Los Angeles, CA) and 3M (St. Paul, MN) respectively. Ethanol (200 proof) and glass slides (Gold Seal Cover glass 24 mm × 40 mm) were purchased from Fisher Scientific (Fair Lawn, NJ). (3-Mercaptopropyl) trimethoxysilane (3-MPS, CN: 175617), dimethyl sulfoxide (DMSO), lyophilized bovine serum albumin (BSA, CN:A2153), Silica gel Rubin drying bags (CN: 72811), and D-(+)-Trehalose dihydrate (Trehalose, CN:T9531) were obtained from Sigma-Aldrich Corporation (Saint Louis, MI). N-g-Maleimidobutyryloxy succinimide ester (GMBS), and NeutrAvidin protein were obtained from Pierce Biotechnology (Rockford, IL). Phosphate buffered saline (PBS) was purchased from Gibco (Grand Island, NY). A 4’-6-diamidino-2-phenylindole (DAPI) was obtained from Invitrogen (Carlsbad, CA). Biotinylated anti-CD4 antibody (Clone 13B8.2, CN:COIM0704) was purchased from Fisher Scientific (Fair Lawn, NJ). Alexa Flour 488 (AF488) conjugated Anti-CD4 antibody (Clone RPA-T4, CN: 557695) and BD FACS Lysing Solution (CN: 349202) were purchased from BD Bioscience (Becton, Dickinson and Company, San Jose, CA). 1X RBC Lysis Buffer (CN: 00-4333-57) was purchased from eBioscience (San Diego, CA). FoodSaver® Bags - 6 × 9” (CN:S-19138) was obtained from Uline (Pleasant Prairie, WI).

### Microfluidic device fabrication and surface functionalization

The microfluidic channels were designed to be compatible for manual pipetting and fabricated without using a cleanroom and lithography. In each microfluidic device, three parallel channels (dimensions: 25 mm × 4 mm × 80 μm) were created in DSA using laser cutter (Versa Laser™, Scottsdale). Inlet and outlet holes (0.65 mm diameter) were cut in PMMA using laser cutter (Versa Laser™, Scottsdale). DSA and PMMA were then assembled together. Glass cover slide was cleaned with 70% ethanol in distilled (DI) water and dried by nitrogen gas. Glass cover slide was then treated with oxygen plasma (100 W, 1% oxygen) for 2 minutes in a PX-250 chamber (March instruments, Concord, MA) to form the hydroxyl (OH) surface functional groups followed by 30 minutes incubation with silanizaton solution (4% (v/v) 3-MPS in ethanol) in a petri dish at room temperature for covalent binding. After incubation cover slide was washed with ethanol and was let to dry for 3–4 minutes at room temperature. Microfluidic device was assembled by sandwiching DSA between PMMA and cover slide (Fig. 1a).

Channels were washed 3 times with PBS and GMBS solution (4% (w/v) GMBS dissolved in 10% DMSO in PBS) was pipetted into microfluidic channels. Devices were incubated for 30 minutes at room temperature. From now onwards, channels were washed 3 times with PBS after each incubation step. Then NeutrAvidin solution (0.1 mg/mL in PBS) was pipetted and devices were incubated for 2 hours at 4 °C. For capturing CD4 T cells, biotinylated anti-CD4 antibody (20 μg/mL in PBS (Supplementary Table 1)) and 1% (w/v) BSA in PBS (to block unspecific binding) was incubated for 30 minutes at room temperature sequentially. To preserve the immobilized functional group 0–5% (w/v) Trehalose in DI water was pipetted and incubated for 30 minutes. Then the microfluidic devices were ready for drying and preservation.

### Drying channels, packing and reactivation of devices

We tested four methods for drying of microfluidic devices. First method is placing devices on standard hot plate at 37 ^o^C, second method is applying −761 mmHg vacuum inside an oven (Isotemp® vacuum oven, Model 280A, Fisher Scientific Inc), third method is applying 37 ^o^C temperature at −761 mmHg vacuum inside an oven, and the fourth method is centrifuging devices at 6600 rpm for 5 seconds using a benchtop centrifuge (Fisher Scientific Mini Centrifuge, CN: 05-090-100) to remove excessive fluid present inside the channels followed by applying 37 ^o^C temperature at −761 mmHg vacuum inside vacuum oven. This mini centrifuge takes about 5 seconds to accelerate to 6600 rpm and 5 seconds to stop, hence, the total centrifugation time is 15 seconds. During centrifugation step, devices are placed with inlets facing down. These four methods are compared for drying efficiency by taking images of devices at every 30 minute interval. Device images are taken using a cell phone camera. To compute the ratio of dried versus wet surface area of the channels, images are processed by *ImageJ* software (National Institute of Health, http://rsbweb.nih.gov/ij/). Device images were converted into greyscale using *ImageJ* threshold function. Ratio of dried versus wet area was calculated by using ‘analyze and measure’ tab. After drying, the devices are vacuum sealed together with silica gel drying bags using FoodSaver® Vacuum Sealer H-340 Uline (Pleasant Prairie, WI). The bagged devices are ready for storing at room temperature. The preserved devices are reactivated using a PBS wash before cell capture experiments. 100 μL of PBS was pipetted into each channel twice to remove trehalose from microfluidic channels.

### CD4 T cell capture, imaging and counting

Fresh whole blood was pipetted into the device channels and incubated for 25 minutes at room temperature. Channels were washed three times to remove any unattached cell. Cells were fixed with 4% (v/v) paraformaldehyde (PFA) solution in PBS for 10 minutes. After fixing, cells were stained with 0.2% (v/v) DAPI solution (DAPI stock solution concentration: 5 mg DAPI in 1 mL of deionized water (Supplementary Table 1)) and 1% (v/v) AF-488 conjugated anti-CD4 (Stock concentration: 100 μg/mL AF-488 in PBS (Supplementary Table 1)) for 90 minutes at 4 °C. After each incubation step the channels were washed 3 times with PBS.

Fluorescent microscope (Carl Zeiss microscope, Jena, Germany) was used for Bright Field and fluorescent imaging. DAPI and AF-488 images of captured were taken with UV (359 nm/461 nm) and CY5 (639 nm/650 nm) filters. DAPI (blue) stained cells indicated the captured white blood cells (WBC, nucleated cells), whereas AF-488 (green) stained cells indicated the captured CD4 T cells. After manual counting of cells, the ratio of CD4 T cells to WBC (green/blue) gave the capture specificity. Capture efficiency was calculated by using the formula Capture efficiency 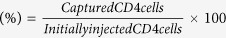.

Initial number of CD4 T cells was determined by dividing AF-488 stained cells in isolated WBC samples. To determine initial CD4 count in whole blood, WBCs were isolated by incubating whole blood with 10% RBC lysis solution for 3 minutes. The cell suspension was centrifuged at 150 g for 5 minutes. Supernatant was removed and the cell pellet was resuspended with same volume of PBS as initial blood volume. The isolated WBCs were fixed with 4% PFA for 10 minutes. Cells were stained with 0.2% (v/v) DAPI solution (DAPI stock solution concentration: 5 mg DAPI in 1 mL of deionized water) and 1% (v/v) 100 μg/ml AF-488-anti-CD4 in PBS solution for 90 min at 4 °C. Between each step, washing was performed by centrifuging the sample at 150 g for 5 minutes following by discarding supernatant and re-suspending the pellet. Finally DAPI stained WBC and AF-488 stained CD4 T cells were counted using haemocytometer under fluorescent microscope.

### Statistical Analysis

At least three channels were tested for each time point and the results of CD4 capture efficiency and specificity were expressed as a mean ± standard deviation. Statistical analysis was performed by one-way analysis of variance (ANOVA). A value of P < 0.05 was considered statistically significant.

### Lensless shadow imaging and automated cell counting

Complementary metal oxide semiconductor (CMOS) sensor (IDS imaging development systems, Germany, UI-1492LE-M) was used for lensless imaging. This 10 megapixel CMOS sensor detects the shadows of captured cells over an area of 6.4 mm × 4.6 mm at a time with a resolution of 3840 × 2748 pixels (pixel size = 1.67 μm). Five images are needed per channel to scan the whole channel area. It requires less than a minute to image all the captured cells inside a channel. A homemade stage is built to hold the CMOS sensor and a microfluidic device in place (Fig. 5). The light emitted by a high brightness white LED (276-0024, RadioShack) passes through transparent PMMA cover and reaches the cells captured on bottom glass cover. If a point light source is placed far from the object, it can be assumed as a planer light. The lensless images of the captured cells are shown in Fig. 5c. Shadow images of the captured cells are processed and counted automatically using a cell detection and counting software programmed by using MATLAB (Mathworks, Natick, MA, USA)^12^. The algorithm for counting cell shadow images are based on event detection using space domain two dimensional cross-correlation. To get a sample shadow cell imaging, 30 cell shadow images are manually obtained and the average of these images became the averaged cell image. Microfluidic channel images were cropped to remove the inlet and outlet holes, and air bubbles from the images. To get a better contrast, an adaptive contrast enhancement by piecewise linear transformation was applied to images (Supplementary Figure 4a,b). Cross correlation of the averaged cell image and the image of cells to be counted was calculated. The areas showing higher correlation values compared to adaptive threshold (value = 1,100,000 (arbitrary units)) were identified as cells. Then peaks are automatically counted to get the total number of cells. This battery operated system composed of CMOS sensor and mobile computer, and can be potentially employed for POC settings.

## Additional Information

**How to cite this article**: Asghar, W. *et al.* Engineering long shelf life multi-layer biologically active surfaces on microfluidic devices for point of care applications. *Sci. Rep.* **6**, 21163; doi: 10.1038/srep21163 (2016).

## Supplementary Material

Supplementary Information
