# Supplementary material for: Engineering long shelf life multi-layer biologically active surfaces on microfluidic devices for point of care applications
Source: Sci Rep. 2016 Feb 17;6:21163. doi: 10.1038/srep21163 (PMC4756328; doi:10.1038/srep21163)
Supplement: Supplementary Information [file srep21163-s1.pdf]

Supplementary Information

Engineering long shelf life multi-layer biologically active surfaces on microfluidic devices for point of care applications

Waseem Asghar[1,2,#], Mehmet Yuksekkaya[3,4,#], Hadi Shafiee[3], Michael Zhang[3], Mehmet O Ozen[1], Fatih Inci[1], Mustafa Kocakulak[4], and Utkan Demirci[1,3,*]

[1] Bio-Acoustic-MEMS in Medicine (BAMM) Laboratory,
Department of Radiology, Canary Center at Stanford for Cancer Early Detection, Stanford University School of Medicine, Stanford University, Palo Alto, CA 94304

[2] Present Address: Department of Electrical & Computer Engineering and Computer Science, Florida Atlantic University, Boca Raton FL, 33431

[3] Bio-Acoustic-MEMS in Medicine (BAMM) Laboratory,
Division for Biomedical Engineering, Renal Division, Department of Medicine,
Brigham and Women's Hospital,
Harvard Medical School,
Cambridge, MA, 02139, USA

[4] Başkent University, Faculty of Engineering, Biomedical Engineering Department, Ankara, Turkey

Corresponding Author: E-mail: utkan@stanford.edu
[#] These authors contributed equally to this work.

Supplementary Table 1. Concentrations used for antibodies and stains

Material	Concentration used	Stock concentration
NeutrAvidin	0.1 mg/mL in PBS	1 mg/mL in PBS
Biotinylated anti-CD4 antibody	20 µg/mL in PBS	0.2 mg/mL in PBS
4’-6-diamidino-2-phenylindole (DAPI)	0.2% (v/v) in DI water	5mg/mL in DI water
AF-488 conjugated anti-CD4	1% (v/v) in PBS	100 µg/mLin PBS

Supplementary Table 2. Comparison between actual cell count and CMOS sensor/Matlab algorithm based automatic cell count

Sample	Manual cell count	Correlation algorithm automatic cell count	Difference (%)
1	12326	12646	2.59
2	5366	5467	1.88
3	12326	12225	0.8

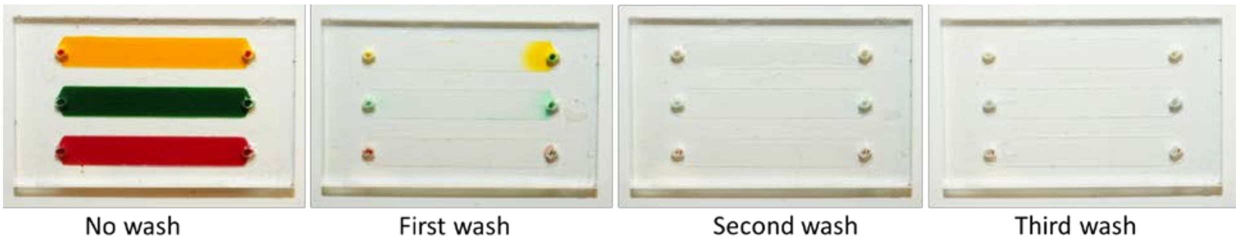

Supplementary Figure 1. Washing of stabilized microfluidic devices. Two different food dye solutions (red and green) and whole blood were injected into microchannels before performing PBS wash steps with manual pipetting. Device images were taken before washing, and after first, second, and third wash with PBS. Channels images indicated that food dyes and blood was removed after washing steps.

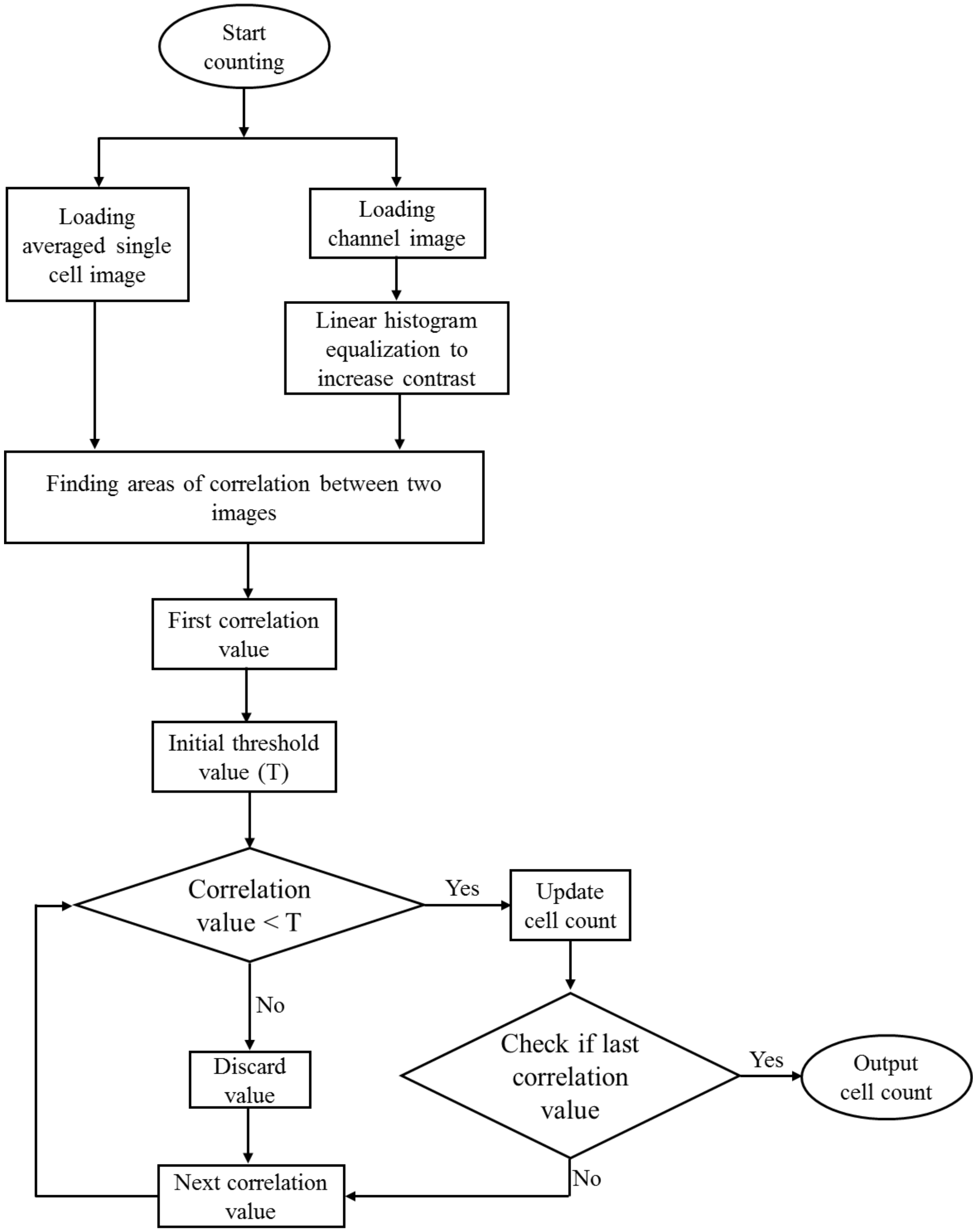

Supplementary Figure 2. Flow chart of cell counting algorithm. Averaged cell image is compared with channel image (image for cell counting), and areas of higher correlation are identified as cells.

(a)

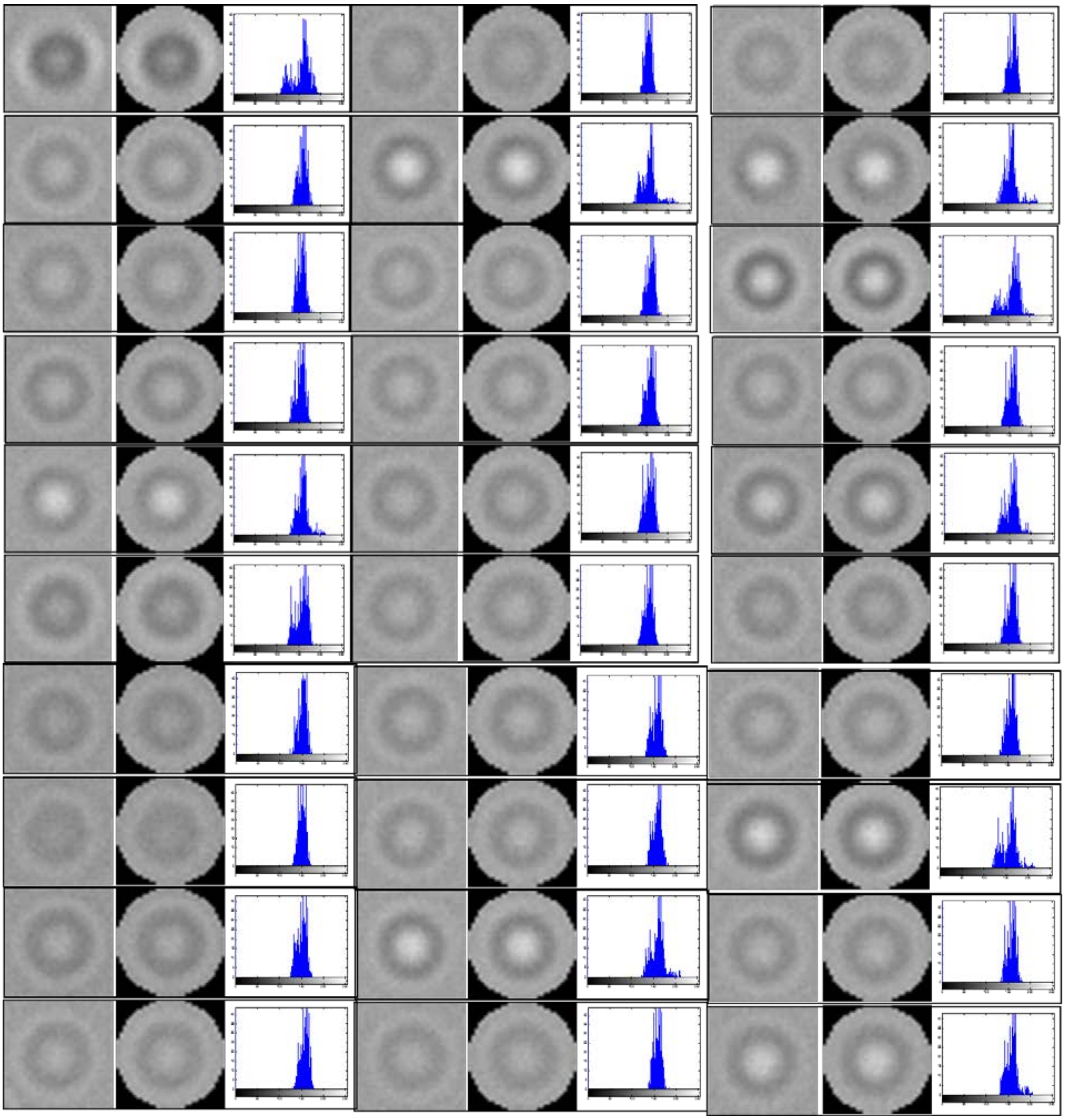

(b)

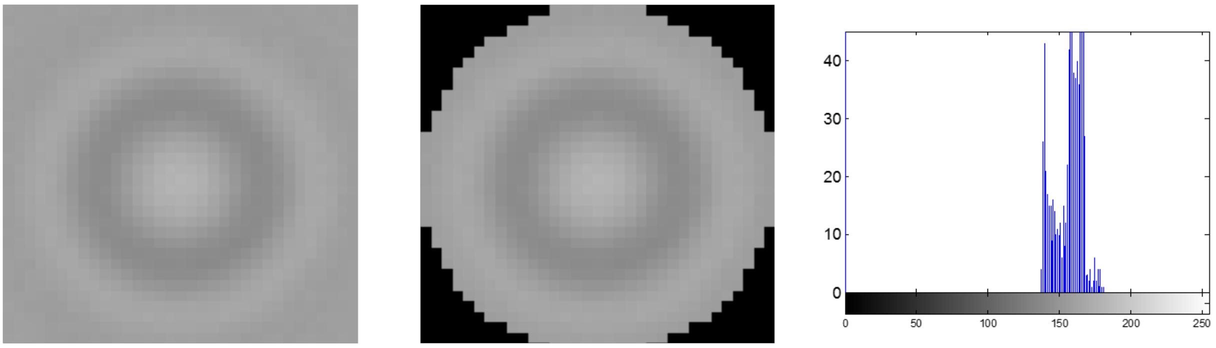

Supplementary Figure 3. Generation of an averaged cell shadow image. (a) 30 different cell shadows and their intensity histograms (right panels) are analyzed. These cell shadows are selected randomly from different areas of a microfluidic channel. Each cell shadow is of 30x30 pixel size. Circular cell shadow areas are selected further by cropping the edges (middle panels) as edges do not contribute to cell shadows. (b) The intensity values from all

30 samples were averaged to generate a single averaged cell image for correlation. The intensity histogram of averaged cell shadow is shown in right panel.

a

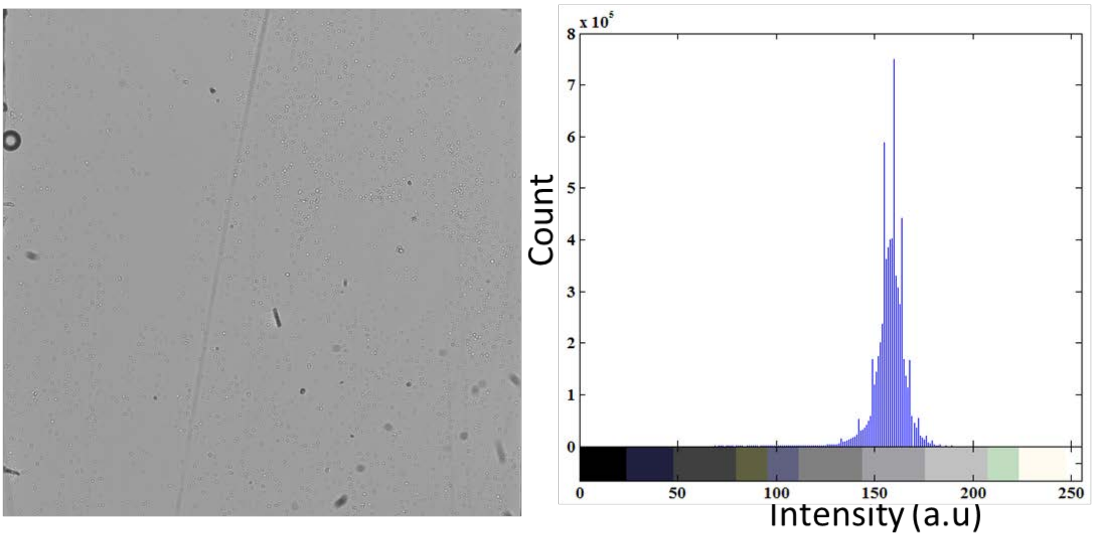
x 10^5
Count
Intensity (a.u)
0
50
100
150
200
250

b

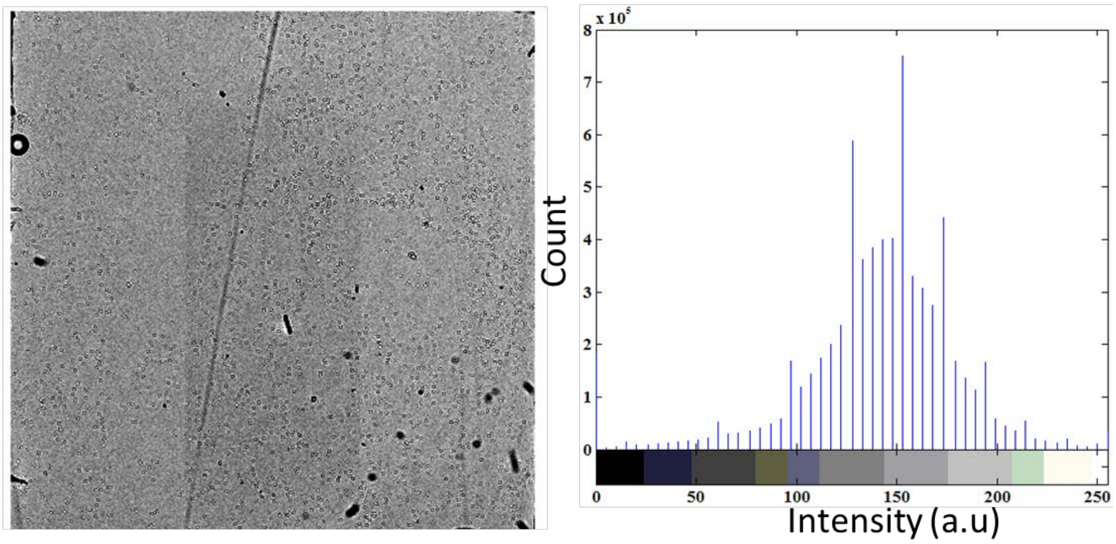
x 10^5
Count
Intensity (a.u)
0
50
100
150
200
250

c

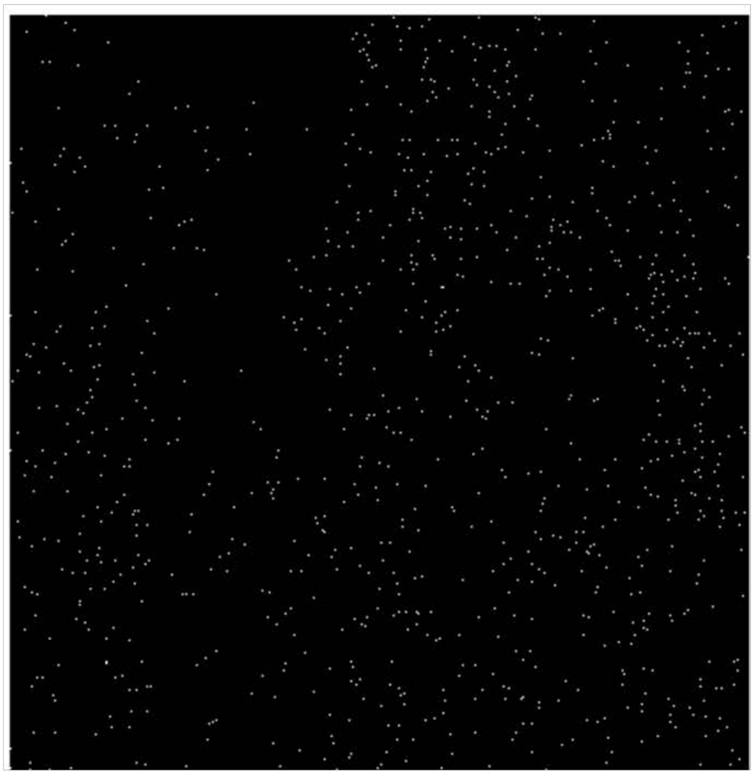

d

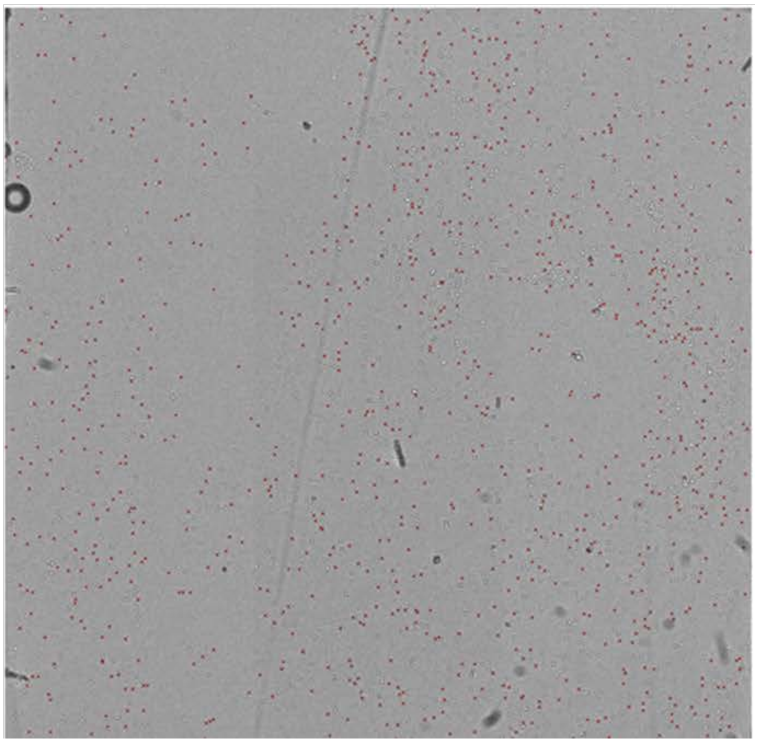

Supplementary Figure 4. Automated counting of captured cells inside a microfluidic channel. (a) Original image of microchannel taken by CMOS sensor and its intensity histogram (right). (b) Image after contrast enhancement and its intensity histogram (right). (c) The cells detected using developed MATLAB algorithm where averaged shadow image is correlated with every cell shadow to determine whether the detected point is a cell or a debris. (d) Image showing cell shadows and detected cells using developed algorithm.

a

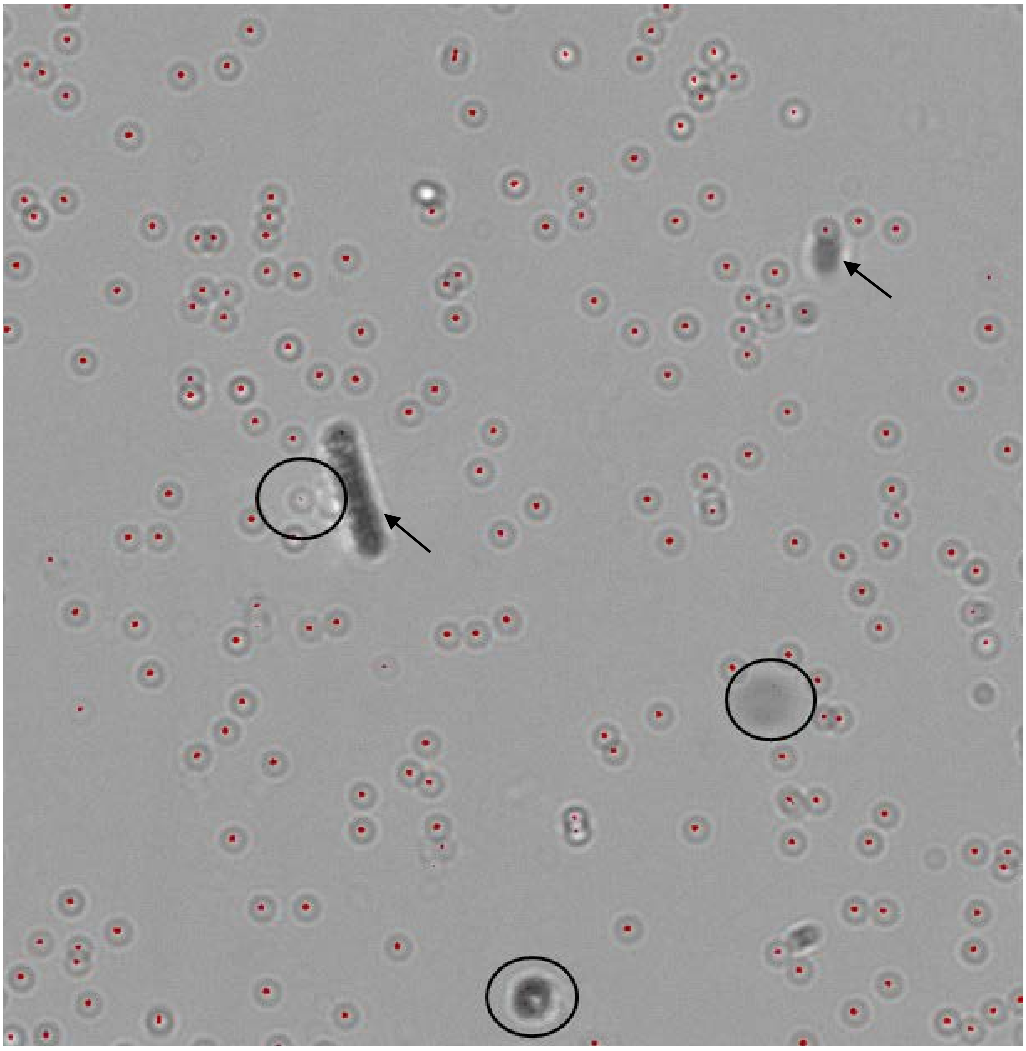

Threshold = 25,000 (arbitrary units)

b

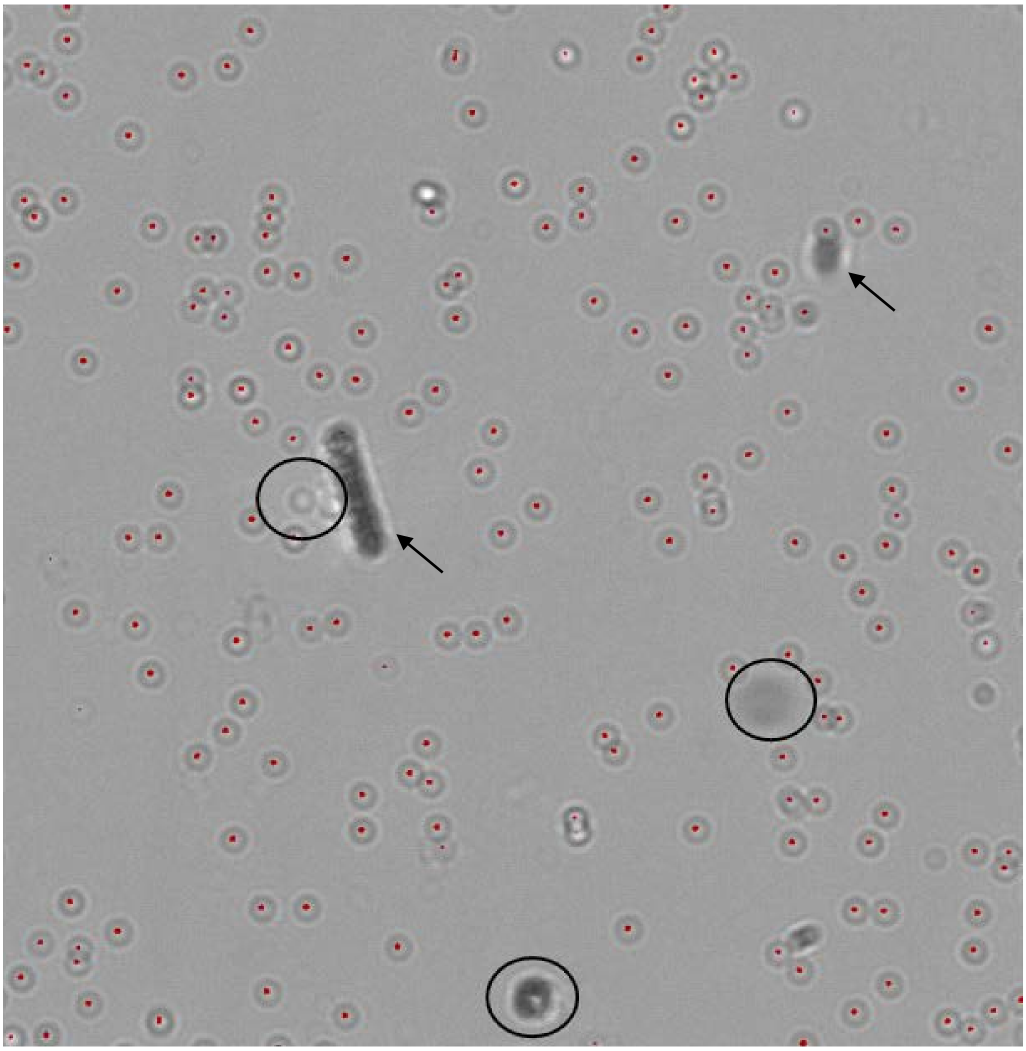

Threshold = 80,000 (arbitrary units)

Supplementary Figure 5. User optimization of threshold value. (a) At lower threshold values (25,000 a.u. in this case), the software identifies some non-cell events as cells as highlighted by black circles. (b) Using optimal threshold values (80,000 a.u. in this case), background noise is reduced by eliminating non-cell events. At both threshold values, software correctly identifies non-cell events which are significantly different from cell shadows, as highlighted by arrow in (a) and (b).
